# Cyanide Content of Cassava Food Products Available in Australia

**DOI:** 10.3390/foods11101384

**Published:** 2022-05-11

**Authors:** Alicia A. Quinn, Harry Myrans, Roslyn M. Gleadow

**Affiliations:** School of Biological Sciences, Monash University, Clayton, VIC 3800, Australia; alicia.quinn@monash.edu (A.A.Q.); harry.myrans@monash.edu (H.M.)

**Keywords:** cassava, cyanide, cyanogenic glucosides, food safety, food processing, konzo, linamarin, lotaustralin, toxicity

## Abstract

In 2009, Food Standards Australia New Zealand set a total cyanide content limit of 10 ppm for ready-to-eat cassava products to address food safety concerns about cyanogenic glucosides in cassava. This study surveys a range of cassava food products available in Melbourne, Australia, ten years after the implementation of these regulations. Of all the products tested, the mean cyanide content was greatest in ready-to-eat cassava chips (48.4 ppm), although imported ready-to-eat products had a higher mean cyanide content (95.9 ppm) than those manufactured in Australia (1.0 ppm). Cyanide was detected in frozen cassava products (grated mean = 12.9 ppm; whole root mean = 19.8 ppm), but was significantly reduced through processing according to packet instructions in both product types. Three methods were used to quantify total cyanide content: the evolved cyanide method, the picrate absorbance method and the picrate chart method, with satisfactory agreement between methods. The picrate absorbance and chart methods reported mean cyanide contents 13.7 ppm and 23.1 ppm higher, respectively, than the evolved cyanide method. Our results reaffirm the need for the ongoing testing of cassava food products, especially ready-to-eat products whose cyanide content will not be reduced before consumption.

## 1. Introduction

Cassava (*Manihot esculenta* Crantz) is a staple crop for over 800 million people throughout Africa, Asia and the Pacific Islands [1], where it is favoured for its ability to produce well in marginal soils, and its tolerance to adverse environmental conditions such as drought [2] and salinity [3]. Cassava is mainly grown for its tuberous roots, which are high in carbohydrates but comparatively low in protein, although its leaves are also edible [4,5]. Its tubers, leaves and stems all contain the cyanogenic glucosides (CNglc) linamarin and lotaustralin. CNglc release hydrogen cyanide (HCN) upon hydrolysis by their degradative enzymes, which are generally spatially separated from CNglc within the plant [6,7]. This process, called cyanogenesis, is traditionally thought to be a defence against herbivory [7].

The presence of CNglc in food products, and the subsequent ingestion of HCN at high levels, can have adverse health effects, including nausea, vomiting, diarrhoea, dizziness and weakness [8,9,10], with a lethal dose of 3–6 mg HCN/kg body weight [11]. Long-term exposure to high levels of HCN can lead to neurological conditions such as konzo and tropical ataxic neuropathy [1,12].

Given the potential risks of eating cassava, effective processing techniques that reduce cyanide content, such as boiling, fermenting and grating, were developed by Indigenous peoples of the Amazon during the domestication of cassava in the region [13]. Similar techniques continue to be used in areas where cassava is regularly consumed [14,15]. However, increasing amounts of cassava are now grown commercially and exported, meaning there is greater availability of cassava products in regions in which they have not traditionally been consumed [16,17,18]. Populations of these regions may not be aware of the risks of consuming inadequately processed cassava products. Therefore, in order to mitigate risks, regulations have been introduced to limit cyanide content in cassava-based foods in some jurisdictions. In Australia, a 10 ppm (10 mg/kg FW) limit of total cyanide was introduced by Food Standards Australia New Zealand (FSANZ) in 2009 for ready-to-eat (RTE) cassava chip products [19], matching the limit recommended by the World Health Organization [20]. Total cyanide refers to the sum of cyanide present in tissues either in CNglc or as free cyanide. Other cassava products, such as frozen root tubers, are also available in Australia, but are subject to a limit of 50 ppm total cyanide—the standard for low-cyanide ‘sweet’ cassava varieties [21].

Previous studies have investigated the presence of CNglc in cassava products on the market in Australia [17,22], but in the case of frozen products, they have not looked at how food preparation methods affect their toxicity. This study aims to provide an updated understanding of cyanide content in cassava-based food products available in Melbourne, Australia, 10 years after the implementation of the FSANZ limit of 10 ppm total cyanide in cassava chip products. We measured total cyanide content using three methods: the semiquantitative picrate chart method, the picrate absorbance method and the evolved cyanide method. This allowed us to compare the measurements of the two picrate methods, which are commonly used in developing countries [23], with those of the highly accurate evolved cyanide method [24,25,26]. FSANZ encourages food producers to monitor their products, but currently does not mandate which cyanide content analysis method is used [19].

## 2. Materials and Methods

### 2.1. Product Survey

Cassava-based food products were sourced from supermarkets and specialty stores around Melbourne, Australia, in July 2018. Products with cassava or tapioca listed as the first or second ingredient were selected [17], including cassava and tapioca chips, tapioca starch, frozen cassava roots and frozen grated cassava (Table S1 in Supplementary Materials). A total of 21 products were selected for this study, with origins in Asia and Oceania. Three replicate packets of each product, displaying the same production code or best before date, were used.

### 2.2. Sample Preparation

Sample preparation was the same for all three cyanide content analysis methods. Chip products were ground with a mortar and pestle and the powder was analysed. Frozen products were thawed at 4 °C before sampling. Whole root pieces were prepared following Bradbury et al. [27]: a 1–2 mm disc was cut from the central part of the root, and from this disc a wedge-shaped sector was removed, cut into 1–2 mm cubes and immediately set up for analysis. Grated products were sampled without further processing, as were flour and starch products.

A subset of the frozen cassava products (four whole root products and two grated products) were used to test the effect of cooking on the cyanide content of cassava products. The cooking method was determined from the preparation instructions on the packaging of frozen cassava products, the majority of which stated to: (a) boil water with salt, and (b) add product and boil for up to 20 min or until soft. Therefore, all products were added to boiling water and simmered for 20 min, drained, blotted dry with paper towel and prepared as above.

### 2.3. Evolved Cyanide Method

The evolved cyanide method was used to measure cyanide content of food products, following Gleadow, Pegg and Blomstedt [3]. Samples were assayed in duplicate or triplicate, with 50 mg weighed into 4 mL scintillation vials before the addition of 300 µL phosphate buffer (0.1 mol/L, pH 6.0) containing 30 µL (*v*/*v*) cassava latex solution (10 mg/mL *w*/*v* in 0.1 mol/L phosphate buffer, pH 6.0). A microtube containing 200 µL 1 mol/L NaOH was placed in the vial before sealing to collect the gaseous HCN as NaCN. Assays were freeze-thawed twice and incubated at 37 °C overnight, after which the microtubes were collected and the resulting NaCN was assayed colorimetrically as previously described [28]. Standard solutions of NaCN (380970, Sigma-Aldrich, St. Louis, MO, USA) were prepared in 0.1 mol/L NaOH for calibration curves (r > 0.99) in the range of 0–200 µM, and content of HCN equivalents for products was calculated by division with sample weight. Cyanide content is reported in relation to fresh weight for all products. Where unspecified, reported results were measured using the evolved cyanide method. Limits of detection and quantitation for our study were estimated from calibration curves to be 0.34 and 1.02 µg CN/mL NaOH, respectively. Mean percent recovery of cyanide using this method was measured at 95.7% by Gleadow [29].

### 2.4. Picrate Methods

Cyanide content was also measured using picrate kits, following Burns, Bradbury, Cavagnaro and Gleadow [17] and Haque and Bradbury [30]. Samples were assayed in duplicate. Then, 100 mg of product was placed in a vial containing a paper disc infused with linamarase, 1 mL of phosphate buffer (1 mol/L, pH 6.0) and a strip of picrate paper, tightly capped and incubated at 30 °C for 24 h. The picrate paper was then removed and compared to the chart [23] (picrate chart method). The picrate paper was then eluted for 30 min with 5 mL of distilled H_2_O, with absorbance of the eluted solution measured at 510 nm in a Varian Cary 50 UV-Vis spectrophotometer (Agilent Technologies, Santa Clara, CA, USA), and cyanide content calculated as HCN equivalents (ppm) by multiplying absorbance by 396 [23,27] (picrate absorbance method). A negative control containing no food product and a positive control containing a 20 µL sample of 50 ppm linamarin were measured alongside the food samples for both picrate methods. Mean percent recovery of cyanide from linamarin using the picrate absorbance method has previously been measured at 102% [30].

### 2.5. Statistical Analysis

Within product types, differences in cyanide content measurements among the different methods were analysed by one-way repeated measures ANOVA. The effect of cooking on cyanide content in cassava root products was analysed using paired t-tests. All analyses were performed using GraphPad Prism Version 8.0.2 (GraphPad Software, San Diego, CA, USA).

## 3. Results and Discussion

### 3.1. Some Ready-to-Eat Products Exceeded the Recommended Cyanide Content Limit

**Figure 1:**
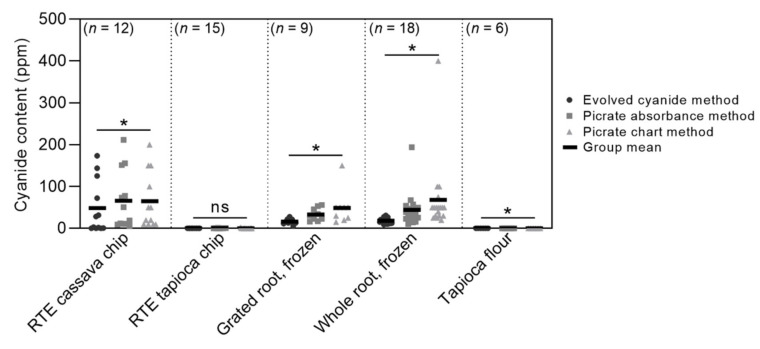
Total cyanide content of cassava products as determined by the evolved cyanide, picrate absorbance and picrate chart methods. All replicates are shown, with group means represented by the black lines. Within product types, differences among cyanide content measurements using the different methods were analysed by one-way ANOVA: * *p* < 0.05; ns = not significant.

Cyanide was detected in all cassava-based product categories (chips and frozen products), while a negligible cyanide content was detected in highly processed tapioca-based products (tapioca chips and flour) (Figure 1; Table 1).

Of the product categories tested, RTE cassava chips contained the highest mean cyanide content (48.4 ppm; SD = 64.3), with one product’s mean cyanide content being as high as 147.7 ppm. These results are consistent with previous studies [17,22] (91 ppm [SD = 106] and 64.2 ppm [SD = 27.5], respectively) for RTE cassava chips on the Australian market. However, both of these previous studies sampled products before the implementation of the 10 ppm limit. Burns, Bradbury, Cavagnaro and Gleadow [17] did report a drop in the mean cyanide content of RTE cassava chips to 7 ppm one year after the implementation of the limit, but our results indicate that this drop was not necessarily continued in the following years.

In this study, all RTE cassava chips manufactured in Australia had s negligible cyanide content (mean = 1.0 ppm, SD = 1.2, *n* = 6), while all imported RTE cassava chips had a cyanide content above the 10 ppm FSANZ limit (mean = 95.9 ppm, SD = 60.8, *n* = 6). This 10 ppm limit for cassava flour and RTE products [19] has been formally adopted in only a few countries, including Australia. Other countries have adopted alternative limits, including Indonesia, where 40 ppm is the limit for cassava flour [31]. The differences in regulatory practices internationally may lead to variation in the cyanide content of imported cassava products in Australia.

All the tapioca-based chips studied contained negligible cyanide content (mean = 0.2 ppm, SD = 0.3), as did the tapioca flour products (mean = 0.2 ppm, SD = 0.1). The highest cyanide content within these product groups was 1.3 ppm, recorded in a tapioca chip product. The extensive processing of cassava to obtain tapioca removes most CNglc [14], and a low cyanide content has been reported for tapioca-based products previously [17,32].

### 3.2. Cooking Reduced Cyanide Content of Cassava Root Products

In the trial comparing cyanide analysis methods, whole root and grated cassava products had mean cyanide contents of 18.5 ppm and 16.3 ppm, respectively (Table 1). Using the evolved cyanide method, all frozen products tested had cyanide content below 50 ppm—the frozen ‘sweet’ cassava limit in Australia [21]. However, five samples across three products were found to exceed 50 ppm when tested with both the picrate absorbance and picrate chart methods, ranging from within 10 ppm to more than 350 ppm above the limit (Figure 1). Because the peel, which contains the majority of CNglc present in tubers [33], had been removed prior to packaging, all HCN detected originated from the parenchyma tissue. The higher cyanide content in some products could indicate some use of the reportedly more hardy ‘bitter’ (high HCN) cassava varieties, or crops that have been exposed to stressful conditions, such as drought, which can cause a higher cyanide content in cassava roots [34].

**Figure 2:**
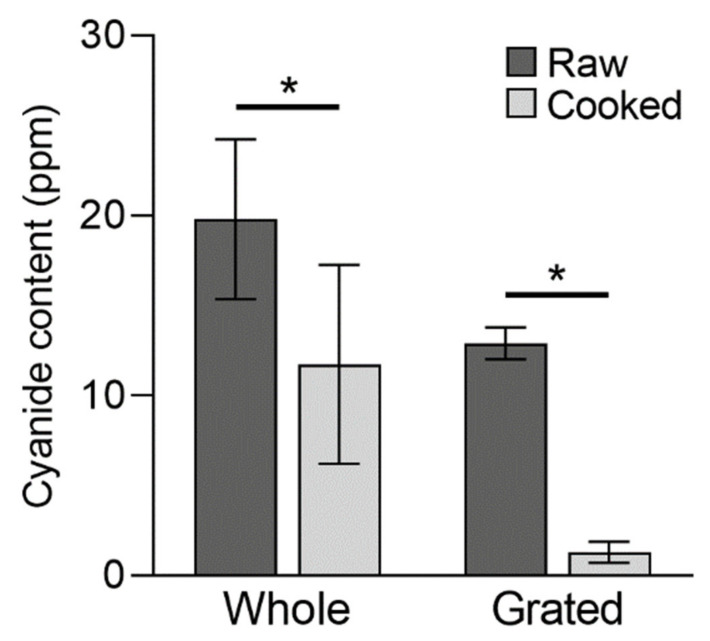
Total cyanide content of whole root (*n* = 12) and grated (*n* = 6) frozen cassava products when raw and cooked, measured using the evolved cyanide method. Values are means ± SEM. Asterisks indicate significant differences (*p* < 0.05) in cyanide content before and after cooking.

In the cooking trial using a subset of the frozen products, cooking significantly reduced the cyanide content of whole root frozen cassava products (t = 2.7; df = 11; *p* < 0.05), with the mean cyanide content decreasing from 19.8 ppm to 11.7 ppm (Figure 2). This result is similar to that observed by Nambisan and Sundaresan [15], who reported a 50% reduction in cyanide content in cassava roots after boiling. Cooking also significantly reduced the cyanide content of grated cassava products from 12.9 ppm to 1.3 ppm (t = 19.8, df = 5, *p* < 0.001), and reduced cyanide content to below the 10 ppm safe limit in all such products (Figure 2).

While boiling is not the most effective processing method for reducing cyanide content in cassava, it is reported to be more effective than baking or frying [14,35]. The variability in cyanide content was greater for the whole roots than grated products both before and after cooking, and cooking did not reduce cyanide content below the recommended 10 ppm standard for ready-to-eat food in some whole root products. This difference between product types may result from the greater processing undertaken in the production of grated cassava, which is known to reduce cyanide content [14,15], and could also be related to the smaller size of grated particles, as boiling is more effective at removing CNglc from smaller-sized cassava pieces [15].

The fact that cyanide content in some whole root products was not reduced below 10 ppm when cooked according to the packet instructions, despite the frozen samples complying with the 50 ppm standard, suggests that these guidelines should be updated to ensure that these products are safe to eat when fully processed, possibly by either increasing the recommended cooking time or including additional steps. Traditional processing methods to facilitate the removal of CNglc, such as soaking, rinsing or cutting whole roots into smaller pieces, are likely unknown to many consumers.

### 3.3. Cyanide Content Measurements Differed among Methods

There was satisfactory agreement between the total cyanide content measurement methods used in this study. However, the results did differ significantly among the methods in four out of the five product categories (Figure 1; Table 1). On average, the picrate absorbance method returned values 77% (13.7 ppm) higher and the picrate chart method returned values 130% (23.1 ppm) higher than the evolved cyanide method. These differences are much larger than those reported by Djazuli and Bradbury [31], who reported a 17% difference between values measured using another method, acid hydrolysis, and the picrate absorbance method.

The differences in cyanide measurements among the methods account for the several frozen cassava samples that were detected above the 50 ppm ‘sweet’ cassava limit, and likely indicate that these products do not exceed the limit, as was found using the more accurate evolved cyanide method. Importantly, cyanide content was often slightly overestimated by the picrate methods in our study, but rarely underestimated (Figure 1), implying that these simple methods remain useful, prudent tools for estimating cyanide content.

## 4. Conclusions

This study surveyed the cyanide content of a range of cassava and cassava-based products available in Australia 10 years after FSANZ implemented the 10 ppm safe limit for cyanide in ready-to-eat cassava chips. Our findings highlight the need for the ongoing monitoring of cyanide content in cassava-based food products, especially those in the ready-to-eat category. We also note the importance of increasing awareness of the need to adequately process frozen cassava products, which could be accomplished through the revision of cooking instructions on packaging.

## Figures and Tables

**Table 1:** Total cyanide content (ppm) from the cassava-containing products determined by three different methods. Mean (SD) values were calculated using all observations of each product type. Within product types, differences among cyanide content measurements using the different methods were analysed by one-way ANOVA, with F values presented. Asterisks depict significant F values (*p* < 0.05).

Product Type	No. of Products	No. of Replicate Samples	Mean Cyanide Content (ppm)			F Value
			Evolved Cyanide Method	Picrate Absorbance Method	Picrate Chart Method	
RTE cassava chip	4	12	48.4 (64.3)	65.7 (70.7)	65.0 (67.8)	5.9 *
RTE tapioca chip	5	15	0.2 (0.3)	0.7 (0.9)	0.3 (0.9)	2.0
Grated root, frozen	3	9	16.3 (6.5)	33.1 (15.2)	48.9 (40.5)	5.0 *
Whole root, frozen	6	18	18.5 (6.7)	44.0 (40.7)	68.1 (86.0)	5.7 *
Tapioca flour	2	6	0.2 (0.1)	0.1 (0.1)	0.0 (0.0)	10.3 *

## Data Availability

The data presented in this study are available in article.

## Supplementary Materials

The following supporting information can be downloaded at: https://www.mdpi.com/article/10.3390/foods11101384/s1. Table S1: Products tested for cyanide content, with details of the number of packets used in analyses, and the origin, mass and ingredients contained in the products.
