# Supplementary material for: Cyanide Content of Cassava Food Products Available in Australia
Source: Foods. 2022 May 11;11(10):1384. doi: 10.3390/foods11101384 (PMC9141144; doi:10.3390/foods11101384)
Supplement: Supplementary file 1 [file foods-11-01384-s001.zip › foods-1671114-supplementary.pdf]

Supplementary materials

Table S1. Products tested for cyanide content, with details of the number of packets used in analyses, and the origin, mass and ingredients contained in the products.

**Product**	**No. of packets**	**Origin**	**Mass (g)**	**Ingredients**
RTE cassava chips	3	Malaysia	120	**Cassava**, vegetable oil (palm oil), maltodextrin, salt, sugar, yeast extract and flavour enhancers (E631, E627, E363)
RTE cassava chips	3	Indonesia	180	**Cassava**, palm oil (contains antioxidant TBHQ), sugar, grilled cheese, seasoning powder (contains MSG, disodium guanylate, disodium inosinate), salt
RTE tapioca chips	3	Singapore	100	Prawn, **tapioca**, flour, palm oil, salt, MSG
RTE tapioca chips	3	Singapore	70	**Tapioca flour**, palm oleic oil, fresh prawns, wheat flour, sugar, bbq seasoning (sugar, maltodextrin, garlic, onion, chili, paprika, salt, natural flavors, flavor enhancer (E621, E627, E631), tomato, hydrolyzed vegetable protein, food acid E330, yeast extract, caramel coloring, silicon dioxide e551), salt, MSG
RTE tapioca chips	3	Philippines	100	Cornstarch, **tapioca starch**, vegetable oil, (may contain palm or coconut oil), sweet corn flavor (artificial and natural), MSG (E621), salt, sugar, whey powder, maltodextrin, silicon dioxide (E551), antioxidant (E319)
RTE tapioca chips	3	Philippines	50	Wheat, **tapioca starch**, vegetable oil (may consist of coconut oil and/or palm olein), corn starch, salt, sodium diacetate (E262ii), sugar, artificial flavors, MSG (E621) (as flavour enhancer), citric acid (W330), spices, sodium citrate (E331ii), fish sauce, hydrolysed soy protein, lactic acid (E270), FD&C yellow no. 5 (E102), disodium inosinate (E631), disodium guanylate (E627), tertiary butylhydroquinone (E319) antioxidant (added to oil to retard rancidity)
RTE tapioca chips	3	South Korea	75	**Tapioca flour**, palm oil, shrimp (13%), wheat flour (8%), sugar, salt, onion powder, garlic powder
RTE cassava chips	3	Australia (local and imported ingredients)	100	**Cassava** (60%), **tapioca flour**, sunflower oil, sugar, shallot leaf, salt, sesame seeds
RTE cassava chips	3	Australia (local and imported ingredients)	100	**Cassava**, **tapioca starch**, sunflower oil, leek, chia seed, sugar, chili, basil seed, salt, galanghal, pepper, seasoning: 8% (maltodextrin: (maize), salt, rice flour, plant fibre, spices & spice extracts, sugar, acidity regulator (citric acid), vegetable extract, rosemary extract)
Tapioca starch	3	Thailand	500	**Manioc** (88%), water (12%)
Tapioca starch	3	Thailand	454	**Tapioca**, water

Cassava roots - frozen, grated	3	Fiji	1000	**Yellow cassava**
Cassava roots - frozen, grated	3	Fiji	1000	**Cassava**
Cassava roots - frozen, grated	3	Vietnam	5000	**Cassava**
Cassava roots - frozen, whole	3	India	1000	**Tapioca**
Cassava roots - frozen, whole	3	Fiji	1000	**Peeled cassava**
Cassava roots - frozen, whole	3	Fiji	1000	**Cassava**
Cassava roots - frozen, whole	3	Tonga	1000	**Cassava**
Cassava roots - frozen, whole	3	Vietnam	1000	**Cassava**
Cassava roots - frozen, whole	3	Vietnam	1000	**Cassava**
